# Next Generation BTK Inhibitors in CLL: Evolving Challenges and New Opportunities

**DOI:** 10.3390/cancers15051504

**Published:** 2023-02-27

**Authors:** Anna Maria Frustaci, Marina Deodato, Giulia Zamprogna, Roberto Cairoli, Marco Montillo, Alessandra Tedeschi

**Affiliations:** ASST Grande Ospedale Metropolitano Niguarda, Niguarda Cancer Center, Piazza Ospedale Maggiore 3, 20162 Milano, Italy

**Keywords:** acalabrutinib, zanubrutinib, pirtobrutinib

## Abstract

**Simple Summary:**

Chronic lymphocytic leukemia (CLL) treatment scenario is rapidly evolving. As a consequence of longer observation, despite remarkable clinical results, treatment with ibrutinib is associated with long-term toxicities and resistance. New strategies based on BTK inhibition are under development, offering effective salvage treatment both to intolerant and refractory patients. This review is aimed at summarizing and discussing the role of next-generation BTK inhibitors in CLL.

**Abstract:**

Ibrutinib revolutionized the CLL treatment approach and prognosis demonstrating its efficacy and safety even at extended follow-up. During the last few years, several next-generation inhibitors have been developed to overcome the occurrence of toxicity or resistance in patients on continuous treatment. In a head-to-head comparison of two phase III trials, both acalabrutinib and zanubrutinib demonstrated a lower incidence of adverse events in respect to ibrutinib. Nevertheless, resistance mutations remain a concern with continuous therapy and were demonstrated with both first- and next-generation covalent inhibitors. Reversible inhibitors showed efficacy independently of previous treatment and the presence of BTK mutations. Other strategies are currently under development in CLL, especially for high-risk patients, and include BTK inhibitor combinations with BCl2 inhibitors with or without anti-CD20 monoclonal antibodies. Finally, new mechanisms for BTK inhibition are under investigations in patients progressing with both covalent and non-covalent BTK and BCl2 inhibitors. Here we summarize and discuss results from main experiences on irreversible and reversable BTK inhibitors in CLL.

## 1. Introduction

The continuous improvement in the understanding of chronic lymphocytic leukemia (CLL) biology over the past decade has led to the development and introduction of several targeted drugs, with demonstrable advances in the prognosis of this incurable condition.

Bruton tyrosine kinase (BTK) is a cytoplasmic, nonreceptor tyrosine kinase from the tyrosine kinase expressed in hepatocellular carcinoma (TEC) kinase family that is primarily expressed in hematopoietic cells, particularly in B lymphocytes, but not in T cells or plasma cells.

BTK acts as a mediator in the B-cell receptor (BCR) signaling cascade, being involved in B-cells’ proliferation, differentiation and survival. Aberrant BCR signaling pathways contribute to the pathogenesis of B-cell malignancies and are therefore an important target in the treatment of inflammatory reactions and autoimmune diseases, as well as of B-cell malignancies, including CLL [1,2,3].

Irreversible BTK inhibitors (BTKi) covalently bind to cysteine 481 in the ATP-binding pocket of BTK, while reversible BTKi bind BTK non-covalently and do not require C481 to be present [4]. 

In the last decade, the first-in-class covalent BTK inhibitor ibrutinib has shown remarkable activity both in previously treated and untreated CLL patients, thus progressively replacing traditional chemotherapy-based regimens [5,6,7,8,9].

Nevertheless, the off-target inhibition of kinases other than BTK is responsible for several side effects during ibrutinib treatment leading to a proportion of patients discontinuing (16–24%) or reducing treatment (13–23%) due to toxicity [5,6,7,8,9,10,11]. 

Disease progression represents the main reason for ibrutinib discontinuation in 4–37% of patients on ibrutinib indefinite administration [5,6,7,8,9]. Acquired mutations in BTK or in its downstream mediator PLCG2 have been identified in the majority (80%) of ibrutinib-resistant cases [12].

The newer covalent agents, zanubrutinib and acalabrutinib, have shown increased selectivity toward BTK, being potentially able to reduce all the off-target effects that are commonly seen with ibrutinib. On the other hand, non-covalent BTKi, such as pirtobrutinib, are currently in pipeline for approval in CLL and may represent an effective strategy to overcome resistance, binding at alternative sites to the C481 residue bound by covalent binding agents. 

## 2. Next-Generation Covalent BTKi: Efficacy and Tolerability

### 2.1. Acalabrutinib

Acalabrutinib is a potent and selective BTKi, for which ACP-5862 has been identified as the major pharmacologically active metabolite in plasma. The mean exposure of ACP-5862 is approximately two-fold higher than that of acalabrutinib [13]. ACP-5862 is approximately half as potent as acalabrutinib for BTK inhibition and has a similar kinase selectivity profile [14]. Clinical drug interaction studies with itraconazole and rifampicin have demonstrated that acalabrutinib is a sensitive substrate of CYP3A. Nevertheless, when both the parent drug and active metabolite (total active components) are considered for drug-to-drug interactions (DDI), the magnitude of the CYP3A DDI seems to be much less significant [15]. Differently from ibrutinib, results from a physiologically based pharmacokinetic model have demonstrated that no dose adjustment is needed for concomitant administration of acalabrutinib with moderate CYP3A inhibitors [16]. In a population pharmacokinetic analysis on acalabrutinib and ACP-5862, drug solubility was shown to decrease with increasing pH [17]; as a consequence, its absorption greatly depends on gastric pH changes. For the same reason, the use of proton pump inhibitors, associated with a reduction in area under the curve, should be avoided in patients on acalabrutinib [18]. 

In competitive binding assays using a panel of 395 non-mutant kinases, acalabrutinib compared to ibrutinib has shown a greater selectivity towards members of the TEC family of kinases [19]. In particular, some of the ibrutinib off-target kinases such as EGFR, ITK and ERB-B2 receptor tyrosine kinase were not inhibited by acalabrutinib [20,21]. Additional off-target kinases, which also include tyrosine protein kinase, were inhibited by acalabrutinib in vitro at high nanomolar concentrations [21,21].

The activity and safety of single-agent acalabrutinib were first evaluated in 134 R/R patients of a single-arm phase 1/2 trial (ACE-CL-001) [22]. Acalabrutinib was administered orally at different schedules once or twice daily until progression or intolerance. Median of prior lines was 2; unmutated IgHV mutational status (uIGHV) was reported in 75% of patients while 31% carried 17p deletion (17p−). After a median follow-up of 41 months, most patients (56%) remained on treatment; primary reasons for discontinuation were progressive disease (21%) and adverse events (AEs, 11%). Median progression-free survival (PFS) was not reached and estimated 45 months PFS was 62%. Importantly, because of improved trough BTK occupancy with twice-daily dosing, 100 mg bid was determined as the preferred dose for further trials. 

Of note, a cohort of 99 treatment naïve (TN) patients not eligible to chemoimmunotherapy (CIT) was also evaluated in the ACE-CL-001 [23]. After a median follow-up of 53 months, median PFS was not reached and the estimated 4-year PFS rate was 96% (82% in patients with del(17p) and/or mutated TP53, and 91% in patients with complex karyotype).

As acalabrutinib does not inhibit ITK, its reduced interference with antibody-dependent cellular phagocytosis (ADCP) and antibody-dependent cellular cytotoxicity (ADCC) may result in a potential effective combination with anti-CD20 antibodies [24]. In another phase I/II acalabrutinib was administered in combination with obinutuzumab (O-Acala) in 45 patients (19 TN; 26 relapsed/refractory, R/R) [25]. Median observation was 39 and 42 months in TN and R/R, respectively. Overall response rate was 95% in TN and 92% in R/R with up to 32% of the previously untreated patients achieving a complete remission (CR) (8% in previously treated). Median PFS was not reached in both cohorts. This regimen appeared tolerable, with a rate of discontinuation due to AEs (11%) consistent with that of the monotherapy schedule with a similar time of follow-up [22,25].

On this background, two phase III studies demonstrated the superiority of acalabrutinib +/− obinutuzumab over CIT both in relapsed/refractory and previously untreated CLL. 

The multicenter randomized phase III ASCEND trial enrolled 310 patients with ≥1 previous line who received acalabrutinib monotherapy (n = 155) or investigator’s choice (idelalisib plus rituximab, n = 119, or bendamustine plus rituximab, n = 36) [26]. Patients had a median of 2 prior lines (range, 1–10), but prior Bcl-2 or BTK/PI3K inhibitors were not allowed. With up to 4 years follow-up, acalabrutinib significantly prolonged investigator-assessed PFS versus the investigators’ choice arm (42 months PFS 62% versus 19%) with a trend for overall survival (OS) advantage, although more than half of participants enrolled in the comparator arm crossed over to the BTKi. The acalabrutinib PFS benefit was confirmed across all prognostic subgroups. Adverse events led to drug discontinuation in 23% of patients.

In the setting of previously untreated patients, the phase III ELEVATE TN trial randomized elderly/unfit patients (fitness defined by age, CIRS, creatinine clearance) to receive either acalabrutinib alone (n = 179), together with obinutuzumab (O-Acala, n = 179) or obinutuzumab + chlorambucil (O-Chl, n = 177) [27,28]. Regarding biological characteristics, 9% of the patients had del(17p), 18% del(11q), 63% unmutated IGHV and 17% had a complex karyotype. Apart from the expected clear advantage of the two acalabrutinib- arms over O-Chl, interestingly at the 5-year update a higher rate of progression/deaths was observed in the monotherapy versus O-Acala arm (5 years PFS 72% vs 84%, respectively, *p* = 0.0259) with also a trend toward superior OS with the combination. Of note, such PFS difference did not emerge in 17p−/TP53 mutated patients.

All these studies have shown a favorable toxicity profile of acalabrutinib and demonstrated its superiority over both CIT and another BCR inhibitor, idelalisib. Nevertheless, only one trial so far has provided a head-to-head comparison of acalabrutinib with ibrutinib. The phase III ELEVATE RR, in fact, was designed as a non-inferiority study, directly comparing the two BTKis in high risk CLL with 17p and/or 11q deletion [29]. Both agents were given as monotherapy. In this difficult-to-treat population, no differences were reported between the two arms neither for overall response rate (ORR) (acalabrutinib versus ibrutinib 81% versus 78%) nor for PFS (38.4 months in both groups), thus meeting the primary endpoint of the study. 

Differently from the previously treated population, no studies have so far directly compared acalabrutinib to ibrutinib in the setting of treatment naïve. However, at indirect comparison, acalabrutinib given both as monotherapy or combined with obinutuzumab, appears to be associated with a longer, albeit not significant, PFS compared to ibrutinib + obinutuzumab [30,31,32,33].

Differences in AEs between ibrutinib and acalabrutinib are discussed further in the “Safety Evaluation” section.

### 2.2. Zanubrutinib

Zanubrutinib (BGB-3111) is another next-generation BTK inhibitor that has already shown high specificity and oral bioavailability in preclinical studies, with reduced off-target activity in vitro enzymatic and cell-based assay [34]. The drug is rapidly absorbed, with Cmax observed in about 2 h after its oral administration. In the phase I study, the Cmax and the area under the concentration–time curve increased proportionally with the increase of zanubrutinib dosage. Importantly, among the approved BTKi, zanubrutinib seems to be less susceptible to PK modulation by intrinsic and extrinsic factors, thus resulting in consistent, sustained therapeutic exposures [35].

In the phase I, first-in-human AU-003 trial, zanubrutinib was offered as a single agent in 144 CLL/SLL at 40, 80, 160, or 320 mg once daily or 160 mg twice daily [36]. Although median BTK occupancy was comparably high with all dosages in peripheral blood, in lymph node tissues this was more frequently >95% with 160 mg bid than 320 mg once daily, thus leading to the zanubrutinib recommended phase II dose of 160 mg bid.

It is important to highlight that at this dosage, when adjusted for plasma protein binding, zanubrutinib exposure is approximately eight-fold higher than that observed with ibrutinib 560 mg daily [37,38]. Nevertheless, no clear differences in safety or activity were reported between the single or the spliced daily dose.

With a median follow-up of 47.2 months, 123 patients (22 TN and 101 R/R) were evaluated for efficacy. Median prior lines in the previously treated cohort was 2 (range 1–10). About one third of cases in both cohorts had TP53 abnormalities. Median treatment duration was 43 months. ORR was 100% and 95% in TN and R/R patients, respectively. Responses deepened over time reaching about 20% of complete remissions in both cohorts at the 4-year cut-off. Median PFS was not reached in TN and estimated at 61.4 months in R/R. Overall, 46 patients (37.4%) discontinued treatment, mostly due to progressive disease (21% of the whole cohort). Importantly, the occurrence of AEs was the main reason for zanubrutinib definitive interruption only in 9.8% of patients in this series [36].

Comparative phase III studies with zanubrutinib include the SEQUOIA trial [39], conducted in a previously untreated population, and the ALPINE study on relapsed or refractory patients [40]. The SEQUOIA was aimed to compare zanubrutinib (group A) with bendamustine+rituximab (BR, group B) in 479 older or comorbid patients, with PFS as the primary endpoint. Given the well-known lack of efficacy of BR in 17p deleted CLL, 111 patients carrying such aberration were directly assigned to the arm C of the protocol and received zanubruitnib open (group C) [39,41]. With a median follow-up of approximately 2 years, the primary endpoint was met for the cohort comparing zanubrutinib with CIT (hazard ratio 0·42 [95% CI 0·28–0·63]; *p* < 0·0001). Considering all the limitations of indirect comparisons, 86% 2-years PFS with zanubrutinib appears to be consistent with that of ibrutinib (85%) and acalabruitnib (87%) at similar timepoints, and comparable to that of high-risk patients with 17p deletion enrolled in group C (18 months-PFS 88.6%). The PFS results of group C are instead difficult to compare with those of other BTKi due to the small number of patients with TP53 aberrations enrolled in similar first-line trials [5,42], but appear to be comparable to the rate reported at 2 years by Ahn et al. with ibrutinib [43]. The PFS advantage over CIT was present in all risk-subgroups with the exception of IGHV mutated patients, which are those known to benefit most from CIT. As expected from the relatively short follow-up, the possibility to cross over and the presence of an intensive regimen as comparator, no differences in overall survival between zanubrutinib and BR emerged at the last update.

Similarly to that reported above with acalabrutinib, zanubrutinib was also compared head-to-head with ibrutinib in a relapsed/refractory population in the ALPINE study. 

Nevertheless, differently from the patients enrolled in ELEVATE-RR, those in ALPINE were not required to have high-risk CLL (del[11q] or del[17p]). Updates of this trial were recently published by Brown et al. [40]. 

Zanubrutinib met and outperformed the primary non-inferiority endpoint, achieving significantly superior ORR (ORR 78.3% versus 62.5% with zanubrutinib versus ibrutinib, respectively). This difference showed a greater magnitude in 17p deleted patients (ORR 83.3% and 53.8%, respectively). Importantly, ORR in this trial was defined as the rate of partial + complete remissions (PR, CR), and excluded patients achieving PRs with lymphocytosis (PR + Ly). Lymphocytosis is an expected pharmacodynamic event with BTKi, which was shown to be not associated with inferior efficacy or long-term survival outcomes with ibrutinib [38]. Taking this into account, the clinical significance of achieving a deeper response (that excludes the persistence of lymphocytosis) needs be clarified over time.

With a median follow-up of 29.6 months, zanubrutinib showed a significantly longer PFS in respect to ibrutinib (median PFS not reached for zanubrutinib, 34.2 months for ibrutinib, HR: 0.65 [95% CI, 0.49–0.86]; 2-sided *p* = 0.002) both as per investigators and independent reviewer committee assessment. The PFS results favored zanubrutinib consistently across major pre-defined subgroups including IGHV status and patients with del(17p)/TP53. Median OS was not reached in both groups with no statistic differences observed (HR due to death 0.76; 95% CI, 0.51 to 1.11). Finally, compared to ibrutinib, zanubrutinib showed a higher ORR (83.5% versus 74.2%) as per investigators’ assessment, which was consistent with that of the independent review committee.

Differences in safety profile between ibrutinib and zanubrutinib will be discussed in the next section.

Efficacy results on acalabrutinib and zanubrutinib are summarized in Table 1.

### 2.3. Safety Evaluation between Covalent BTKi: Direct and Indirect Comparisons

With 8 years of follow-up data since its initial pivotal study [38], the increasing knowledge on the specific toxicity profile of ibrutinib has improved the early recognition and management of specific AEs, such as supraventricular arrhythmias, hypertension and increased risk of bleeding. Notably, although the incidence of grade ≥3 AEs from ibrutinib initiation tends to decrease while on treatment, the prevalence of some event of special interest remains stable over time, leading to clinically significant rates of discontinuations or dose reduction seen in landmark clinical studies of the BTKi [5,6,7,8,9].

It is important to underline that in several phase III trials ibrutinib was administered in different categories of patients stratified according to age and comorbidities and, even with long follow-up, showed a similar rate of toxicity-related discontinuations (21–24%) across 3 to 8 years of observation [5,6,7,9]. 

However, when focusing on specific adverse events, some difference in toxicity emerged in the elderly. At indirect comparison, in fact, patients enrolled in the ibrutinib + rituximab arm of the ALLIACE 041202 trial (median age, 71 years) [5] showed significantly higher rates of grade 3–5 infections, atrial fibrillation, bleeding and hypertension than young patients receiving the same combination in the ECOG1912 study (median age 58 years) [9].

Next-generation BTKi were developed to maximize the on-target inhibition of BTK with a supposed reduction of adverse events in vivo.

Direct comparisons between BTKi in first-line studies are missing. Long-term data from ELEVATE TN suggest that the addition of obinutuzumab to acalabrutinib does not increase toxicity [28]. 

Despite all the limitations of indirect comparison, when considering two first-line studies analyzed at a similar timepoint, the ILLUMINATE (experimental arm: ibrutinib + obinutuzumab) and the ELEVATE TN trials [6,28], at 45- and 46.9-months follow-up, respectively, the safety profile appears to favor acalabrutinib-based treatments over ibrutinib. In particular, AF, reported in 3.9 and 6% with acalabrutinib arms, was recorded in 15% of patients randomized to ibrutinib + obinutuzumab in the ILLUMINATE study. More important, the discontinuation rate for toxicity in patients treated with acalabrutinib in the ELEVATE TN is almost half that of patients receiving ibrutinib + obinutuzumab (12.3–12.8% versus 22%) or even of those treated with single-agent ibrutinib in the 5-year follow-up of the RESONATE-2 trial (16%) [45].

Differently from the first-line treatments, reliable data on the direct comparison of ibrutinib versus acalabrutinib are provided by the ELEVATE RR trial in previously treated patients.

According to study results, overall fewer patients receiving acalabrutinib versus ibrutinib developed severe adverse events, thus translating to a lower rate of definitive discontinuations due to toxicity (14.7% versus 21.3%, respectively) [29]. The incidence of AF and severe infections was a specific secondary endpoint of the study. While no differences were observed for the infection rate, atrial fibrillation, as well as hypertension, were reported in a significantly lower proportion of patients receiving acalabrutinib versus ibrutinib. Moreover, acalabrutinib was associated with a lower incidence and exposure-adjusted incidence of AF, hypertension and bleeding, which was significant in patients with no prior history of such events.

As for acalabrutinib, only direct comparisons of zanubrutinib with CIT are currently available in first line. The shorter time of observation of zanubrutinib studies does not allow us to draw any comparison with long-term data of the other BTKi in treatment naïve. However, in the phase I zanubrutinib trial, with about 4 years follow-up, rates of toxicity-related discontinuations <10% and AF < 5% seem particularly favorable even in the setting of TN [36].

Again, direct comparison is available in relapsed/refractory population. Treatment management favored zanubrutinib in respect to ibrutinib in the ALPINE trial [40], with a lower number of patients discontinuing therapy in the zanubrutinib arm. Interestingly, atrial fibrillation was the only event of special interest significantly more frequent in patients randomized to ibrutinib versus zanubrutinib (13.3% vs 5.2%), while incidence of hypertension and bleeding were superimposable between the two arms. Similarly, no differences were reported in the rate of severe, serious and fatal adverse. As previously seen in other studies, neutropenia was slightly higher with zanubrutinib, not translating to increased rate of infections. 

Of note, six grade 5 cardiac events occurred during the study, all in the ibrutinib arm. 

Whereas cardiological toxicity appears to be the only relevant factor differentiating the safety profile of the two BTKi and clearly favoring zanubrutinib over ibrutinib, it is possible to speculate that the management of cardiac events might be the event weighing most on the differences observed for treatment discontinuations in this study.

Clinical trials on the use of an alternative BTKi in case of intolerance are available for both acalabrutinib and zanubrutinib [44,46]. Intolerance was defined as the persistence or recurrence of adverse events considered to be related to the first BTKi. Both these phase II studies support the use of an alternative BTKi in case of toxicity, as most patients were able to continue to be treated with an irreversible BTK or presented a recurrence of the adverse event at a lower intensity.

To summarize, any conclusion about the potential advantage of next-generation BTKis in first line should be taken with caution as it comes from cross-trial comparisons and might fail to recognize inherent differences across studies. On the other hand, the availability of two randomized, controlled phase III trials allows a more appropriate comparison of the safety profile of ibrutinib versus next-generation inhibitors in a previously treated population. Compared to ibrutinib, incidence of atrial fibrillation is lower with both acalabrutinib and zanubrutinib and this is probably the event affecting most ibrutinib management. As regards this specific toxicity, zanubrutinib seems to be even more safe than acalabrutinib at indirect comparison. Nevertheless, it should be kept in mind that no definitive conclusions can be drawn so far, given the shorter follow-up (~2–4 years) of zanubrutinib. New onset or worsening of hypertension does not differ between ibrutinib and zanubrutinib, but seems to be significantly reduced in patients receiving acalabrutinib, thus suggesting favoring the use of this inhibitor in this particular setting. 

Further studies on direct comparisons between next-generation BTKi and studies on TN patients are warranted. 

Safety results on acalabrutinib and zanubrutinib are reported and summarized in Table 2.

### 2.4. Other Covalent BTKi

Several other next-generation covalent BTKi are currently under investigation for B-cell malignancies including CLL.

Spebrutinib (CC-292) inhibits BTK by binding irreversibly the same cysteine 481 of ibrutinib [47]. In a phase 1 study on 84 pretreated CLL/SLL patients, including 23.8% with 17p deletion and 21% with 11q deletion, spebrutinib given at different dosages was overall well tolerated and led to a median response duration up to 11 months in the 1000 mg cohort [48]. Although effective even in high-risk cytogenetics, clinical results with spebrutinib are inferior to those already consolidated with ibrutinib, thus resulting in the interruption of its clinical development.

Orelabrutinib (ICP-0229) in vitro showed to be more selective than ibrutinib at 1 µM concentration while targeting BTK with >90% inhibition. The BTKi is currently approved in China for previously treated CLL/SLL [49], following the results of a phase II trial on 80 previously treated patients. In this population orelabrutinib allowed for the achievement of an ORR of 93.8% with an estimated 30-months DOR of 70.6% that was maintained regardless of biologic risk. Of note, no cases of AF or severe hypertension were reported while only one patient developed a treatment-related major bleeding [50].

Tirabrutinib (ONO/GS-4059) as well demonstrated greater selectivity on BTK than ibrutinib. Its activity on B-cell malignancies was reported in 90 R/R patients including 25 CLL. All but one obtained a response within 3 months from treatment start. Low grade diarrhea was the most common AE reported occurring in 18% of patients; hematologic toxicity was the most common grade ≥3 event recorded in CLL, while no cardiac toxicities occurred [51]. Tirabrutinib-based combinations were also analyzed; however, a significant increase of therapy-related toxicities was noted with association to other agents with a reported rate of grade 3 or higher adverse events >70% [52,53].

## 3. Overcoming Resistance: Non-Covalent BTKi

Disease progression is responsible for BTKi discontinuation in 16% to 23% of patients on continuous treatment [8,9,54,55]. Resistance mutations occur in the BTK binding site. Several changes in residue C481 have been reported, while the two most common alterations are cysteine to serine (C481S) or cysteine to arginine (C481R) [56,57]. Cells harboring these mutations cannot be bound by all covalent inhibitors resulting in the need for alternative therapeutic strategies able to overcome resistance. 

Most of the knowledge on resistance comes from ibrutinib experiences; however, similar resistance mechanisms were identified with acalabruitnib [58]. Moreover, Blombery et al. recently described an enrichment in the BTK Leu528Trp mutation of patients on zanubrutinib compared with ibrutinib, opening the door to the possibility of cross-resistance with reversible BTKis [59].

Resistance typically arises in the context of indefinite treatment leading to disease progression and treatment cessation more commonly in pretreated and in patients with TP53 abnormalities [60].

Non-covalent BTKi bind the BTK pocket in a site different from the Cys481 amino acid residue, and thereby they can act both in wild-type and Cys481-mutated patients. Four of these agents were developed in clinical trials and include vecabrutinib, fenebrutinib, nemtabrutinib (MK-1026, formerly ARQ 531), and pirtobrutinib (formerly LOXO-305).

### 3.1. Pirtobrutinib

Pirtobrutinib has marked selectivity for BTK and is able to maintain plasma levels above the BTK IC90 with well-tolerated daily dosing. The first-in-human multicenter phase 1/2 study BRUIN clinical trial was addressed to patients with B-lymphomas including 247 CLL/SLL [61]. No dose-limiting toxicity was reached and 200 mg was selected as phase II dose. Median number of prior lines was 3 (range 1–11). All patients were previously treated with a BTKi, with 77% discontinuing it for refractoriness. Overall, 41% of patients also received a Bcl2 inhibitor. BTK/PLCG2 mutations were found in 92/222 patients (46%). In this heavily pretreated population with no other treatment options, median PFS was 19.6 months (18.2 months in double-refractory population with a median of 5 prior lines). Similar efficacy was observed regardless of BTK mutation status, TP53 aberrations, age and prior treatment with target therapies. Consistently, high ORR were observed across all subgroups. Median time on treatment for the overall safety population was 9.6 months. Toxicity-related discontinuations and dose reductions occurred in 2.6% and 4.5% of patients, respectively. Common BTKi-related adverse events were recorded at a low frequency with pirtobrutinib (AF < 3%; hemorrhages 11.4%; hypertension 9.2%). Most of the adverse events were of grade 1–2; neutropenia was the most common presenting as grade 3–4 in 24.2%. These results support pirtobrutinib clinical development also in early disease stages.

### 3.2. Other Non-Covalent BTKi

Nemtabrutinib was administered in 57 R/R CLL patients enrolled in a phase I/II trial on B-cell malignancies. Overall response rate was 56% in a high-risk population with a median of 4 prior lines including BTKi and Bcl2i in most of cases. Treatment was well tolerated. No difference in responses was reported according to the presence of BTK Cys481S mutation, double refractory status, 17p deletion or IgHV unmutated status [62].

Two non-covalent BTKi, vecabrutinib (SNS-062) and luxeptinib (CG.806), have curbed their clinical development due to suboptimal activity in CLL [63,64].

In a phase I study instead, fenebrutinib presented unexpected toxicity in patients with CLL thus limiting its further development in clinical trials [65].

## 4. Incorporating Next BTK Inhibitors in a Fixed Duration Schedule: Duplets and Triplets

Several factors support the role of a time-limited treatment combination with BTK and Bcl2 inhibitors. These agents, in fact, present a non-overlapping mechanism of action and toxicity profile and act on different compartments. Preclinical studies demonstrated increased functional dependence on Bcl-2 after BTK inhibition [66], potentially leading to deep responses. Importantly, the addition of venetoclax to a BTKi allows for achieving deep responses with the potential of treatment interruption and consequent reduction of adverse events and resistance mutation that are typically seen in continuous therapy.

While ibrutinib-based combinations are the ones later in clinical development, several trials are currently exploring the activity of zanubrutinib or acalabrutinib together with venetoclax with or without anti CD20 MoAb.

A triple combination of acalabrutinib, venetoclax and obinutuzumab (AVO) led to undetectable MRD in 86% of participants, independently of TP53 status. Treatment was safe with a significantly lower rate of AF and hypertension than those previously reported with continuous single-agent acalabrutinib [67]. Given the favorable outcome in high-risks, updated results on the expansion cohort focused on 31 patients with TP53 mutation/17p deletion were recently presented at the 2022 ASH Meeting [68]. As expected in this selected population, additional biologic risk factors were present in this cohort, such as unmutated IgHV in 73.5% and complex karyotype in 26.2%. All patients responded to treatment, including 52% of complete remissions. Rate of uMRD was confirmed >80% both in peripheral blood and bone marrow and remained stable from cycle 16 to cycle 25. At a median follow-up of 35 months, PFS was 93% with only one patient progressing. Hematologic toxicity was the most common reported. AF occurred in 3% of patients; no major bleedings were recorded [68]. Apart from this trial, the AVO combination is currently under investigation in the phase III trial AMPLIFY in non-high-risk patients, in comparison with acalabrutinib + venetoclax and CIT (NCT03836261).

Zanubrutinib combinations are also on study in a frontline setting. The arm D of the above-mentioned SEQUOIA trial consisted of a non-randomized cohort specifically addressed to TN 17p deleted patients [69]. This was an MRD-guided study combining zanubrutinib with venetoclax for 12 to 24 cycles. Treatment was overall well tolerated with only one patient discontinuing the combination due to adverse events and no tumor lysis syndromes were reported. Incidence of neutropenia, an adverse event commonly seen with both the targeted agents, did not increase with the combination (20%).

In this population enriched with high-risk prognostic features (83.3% with complex karyotype, 85.3% with unmutated IgHV, median 17p− percent of abnormal nuclei 77.5%), ORR was 97.2%, including 36% of CR. Notably, the rate of patients with deep response may be underestimated in this trial due to the unavailability of BM evaluations due to COVID-19 restrictions. With a median follow-up of 12 months, one patient progressed and one died due to a secondary malignancy.

Zanubrutinib with obinutuzumab and venetoclax as initial MRD-directed therapy was given in the open label phase II study BOVen [70]. Therapy was discontinued after 8–24 cycles when prespecified uMRD criteria were met. After a median follow-up of 25.8 months, the trial met its primary endpoint with 89% of patients reaching uMRD in both PB and BM, and treatment was stopped after a median of 10 cycles. All patients achieved a response, CR in 57%. Again, severe neutropenia occurred in 18% of patients, not differing from the rate observed with zanubrutinib or venetoclax given as single agents.

Further studies are needed to evaluate if the addition of antiCD20 MoAb to the BTK+Bcl2 combination really gives an advantage and which BTKi should be preferred to use in combination.

## 5. Looking at the Future of BTK Inhibition: BTK Degraders

Several processes involved in the homeostasis of cellular functions, such as proliferation, differentiation and apoptosis, are regulated by the degradation of intracellular proteins.

The regulation of intracellular protein levels depends on a system of ubiquitin-dependent proteolysis that is composed of 3 enzymes: ubiquitin-activating enzymes (E1), ubiquitin-conjugating enzymes (E2) and ubiquitin–protein ligases (E3). The catalyzation of the bond between the ubiquitine polymer and lysine residues of substrate proteins by these intracellular proteins leads to proteosome recognition and protein degradation [71,72].

The use of a proteolysis-targeting chimera (PROTAC), able to selectively degrade intracellular proteins, is emerging as a new potential approach in B-cell malignancies. These molecules are composed of a target protein binding arm, a degradation machinery-recruiting unit and a linker. While the targeting arm binds target proteins, the degradation machinery-recruiting unit links an E3 ubiquitin ligase, thus resulting in the creation of ubiquitin polymers [73]. Considering the potential of these agents to eliminate wild-type BTK or BTKC481S, this therapeutic strategy is under investigation in CLL.

NX-2127 is a first-in-class targeted protein degrader of BTK that in a preclinical work was shown to induce the degradation of both wild-type and mutant BTK [74]. 

Clinical results from a first-in-human phase I study on NX-2127 were recently presented at the last ASH meeting [75]. Overall, 23 CLL patients were enrolled. Median age was 75 years, median of prior lines was 5 (range 2–11). All patients were previously exposed to a covalent BTKi; 78% additionally received a Bcl2 antagonist and one third of the population was previously treated with covalent BTKi, non-covalent BTKi and Bcl2 antagonist. BTK mutations were present in half of patients, while Bcl2 mutations were present in 20%. At median follow-up of 5.6 months, 14/23 patients are still on treatment. In this elderly population with no other available therapeutic options, NX-2127 led to ORR of 33% in 15 evaluable patients. The safety profile in the whole population was consistent with that of BTK-targeted therapies in heavily pretreated patients, mostly grade 3 neutropenia, thrombocytopenia and anemia (36%, 8.3%, 11.1%, respectively) and atrial fibrillation (any grade 16.6%). These data support the use of BTK degraders in double or triple refractory patients, independently from BTK or Bcl2 mutational status.

## 6. Conclusions

Ibrutinib has revolutionized CLL treatment over the years, allowing the successful treatment of even high-risk diseases. Despite these remarkable results, a proportion of patients will discontinue treatment due to toxicity or resistance. Next-generation BTKi have shown to be better tolerated, thus allowing the gain in clinical practice of a significant advantage in terms of treatment management. Furthermore, these agents, with lower rates of cardiovascular effects and bleeding, appear particularly suitable for elderly and unfit patients, which represent the typical CLL population in common practice. Reversible BTKi represent an effective salvage treatment in covalent BTKi-pretreated patients. Unfortunately, as most trial participants received these agents in advanced lines of therapy, remissions are short, especially in double refractory. These patients represent a clear unmet need in CLL. Several trials are currently ongoing aimed to evaluate the efficacy of non-covalent BTKi in earlier phases of disease treatment.

Finally, with the view to reduce toxicity and resistance, time-limited schedules with BTKi plus Bcl2 backbone are appealing options. Doublet and triplet combinations with different classes of agents are currently under evaluation. Although further investigations and longer follow-up are needed to validate the long-term efficacy of these schedules, their introduction in first-line treatment may deeply overturn the CLL concept of therapeutic sequencing in the near future.

## Figures and Tables

**Table 1:** Efficacy results of acalabrutinib and zanubrutinib in clinical trials.

Reference	Schedule	N of pts (Tx Status)	m Prior Lines	17p−/TP53 Mut (%)	ORR (%)	Survival	FU (mo)
**Byrd et al.** **2020** [22]	Acala 100 to 400 mg/d	134 (R/R)	2	23	94	NR Est 45 mo PFS 62%	41
**Byrd et al. 2021** [23]	Acala 100 mg bid or 200 mg/d	99 (TN)	NA	10	97	NR Est 48 mo PFS 96%	53
**Woyach et al.** **2020** [25]	Obi: C1: 100 mg D1, 900 mg D2, 1000 mg D8, D15; C2–6: 1000 mg D1 Acala: 100 mg bid	19 (TN)	NA	22(17p)/ 28(TP53)/ 21(17p+TP53)	95	NR 94.4%	39
**Woyach et al.** **2020** [25]	Obi: C1: 100 mg D1, 900 mg D2, 1000 mg D8, D15; C2–6: 1000 mg D1 Acala: 100 mg bid	26 (R/R)	1	20(17p)/ 25(TP53)/ 12(17p+TP53)	92	NR 72.7%	42
**Sharman et al.** **2022** [27,28]	Acala: 100 mg bid	179 (TN)	NA	12.81	89.9	60 mo PFS 72%	58.2
**Sharman et al.** **2022** [27,28]	Obi: C1: 100 mg D1, 900 mg D2, 1000 mg D8, D15; C2–6: 1000 mg D1 Acala: 100 mg bid	179 (TN)	NA	14	96.1	60 mo PFS 84%	58.2
**Ghia et al.** **2022** [26]	Acala: 100 mg bid	155 (R/R)	1	17.4	83	NR 42 mo PFS 62%	46.5
**Byrd et al.** **2021** [29]	Acala: 100 mg bid	268 (R/R)		37.3	78	PFS: 38.4 mo	40.9
**Rogers et al.** **2021** [44]	Acala: 100 mg bid	60 (R/R)	2	28	64	Est 36 mo PFS 58%	34.6
**Cull et al. 2021** [36]	Zanu 160 mg bid or 320 mg/d or 160 mg/d	101 (R/R)	2	16	95	Est 61.4 mo	43.7
**Cull et al. 2021** [36]	Zanu 160 mg bid or 320 mg/d or 160 mg/d	22 (TN)	NA	16.7	100	NR	54.1
**Tam et al. 2022 (Group A)** [39]	Zanu 160 mg bid	241 (TN)	NA	1	94.6	NR 24 mo PFS 85.5%	26.2
**Tam et al. 2022 (Group C)** [39,41]	Zanu 160 mg bid	111 (TN)	NA	99	90	NR 24 mo PFS 88.9%	30.5
**Brown et al.** **2022** [40]	Zanu 160 mg bid	327 (R/R)	1	22.9	83.5	NR 24 mo PFS 78.4%	29.6

Tx: treatment; FU: follow-up; mo: months; pts: patients; NR: not reported; bid: twice daily; d: daily; Obi: Obinutuzumab; C: cycle; D: day; TN: treatment naïve; R/R: relapsed/refractory; m: median; NR: not reached; NA: not applicable.

**Table 2:** Safety results of acalabrutinib and zanubrutinib and direct comparisons with ibrutinib in clinical trials.

Reference	Schedule	N of pts (Tx Status)	G ≥ 3 AEs (%)	Tox-Related Discontinuations (%)	Tox-Related Reductions (%)	Any Grade AF %, (G ≥ 3)	Any Grade Bleeding (G ≥ 3) %	Any Grade Hypertension (G ≥ 3) %	m Time on tx
**Byrd et al.** **2020** [22]	Acala 100 to 400 mg/d	134 (R/R)	66	13	4	7 (3)	(5)	17.9 (7.5)	41
**Byrd et al.** **2021** [23]	Acala 100 mg bid or 200 mg/d	99 (TN)	35.4 (occurring in >15% of pts)	6.1	NR	5 (2)	66 (3)	22 (11)	41.5
**Woyach et al.** **2020** [25]	Obi: C1: 100 mg D1, 900 mg D2, 1000 mg D8, D15; C2–6: 1000 mg D1 Acala: 100 mg bid	19 (TN) 26 (R/R)	62 77	5.3 15.4	2 pts 1 pt	2 (2)	71 (4)	40 (7)	NR
**Sharman et al.** **2022** [27,28]	Acala: 100 mg bid	179 (TN)	25 events (occurring in ≥30%) + 86 events of special interest	12.3 (at 45.7 mo)	NR	7.3 (1.1)	43.6 (3.6)	8.9 (3.9)	58.1
**Sharman et al.** **2022** [27,28]	Obi: C1: 100 mg D1, 900 mg D2, 1000 mg D8, D15; C2–6: 1000 mg D1 Acala: 100 mg bid	179 (TN)	76 events (occurring in ≥30%) + 98 events of special interest	12.8 (at 46.6 mo)	NR	6.2 (1.1)	49.4 (4.5)	9.6 (4.5)	58
**Ghia et al.** **2022** [26]	Acala: 100 mg bid	155 (R/R)	68	23	6	8 (1)	31 (3)	8 (5)	44.2
**Byrd et al.** **2021** [29]	Acala: 100 mg bid Ibrutinib:420 mg/d	268 (R/R) 265 (R/R)	68.8 74.9	14.7 21.3	13.2 15.2	9 (4.5) 15.6 (3.4)	38 (3.8) 51.3 (4.6)	8.6 (4.1) 22.8 (8.7)	38.3 35.5
**Rogers et al.** **2021** [44]	Acala: 100 mg bid	60 (R/R)	129 events	17	6.7	3.3 (0)	8.3	13.3 (3.3)	32
**Cull et al. 2021** [36]	Zanu 160 mg bid or 320 mg/d or 160 mg/d	22 (TN) 101 (R/R)	73.2	9.8	8.9 (at least once)	4.9 (3.3)	38.2 (3.3)	19.5 (8.9)	43
**Tam et al. 2022 (Group A)** [39]	Zanu 160 mg bid	241 (TN)	53	8	14	3	41 (3.7)	6 (6)	26.4 (m safety FU)
**Tam et al. 2022 (Group C)** [39,41]	Zanu 160 mg bid	111(TN)	55	5	10		46 (5)	5 (5)	30 (m safety FU)
**Brown et al.** **2022** [40]	Zanu 160 mg bid Ibrutinib:420 mg/d	327 (R/R) 325 (R/R)	67.3 70.4	16.2 22.2	12.3 17	6.2 (2.5) 13.3 (4)	42.3 (3.4) 41.4 (3.7)	23.5 (15.1) 22.8 (13.6)	28.4 24.3

Tx: treatment; G: grade; AEs: adverse events; Tox: toxicity; AF; atrial fibrillation; FU: follow-up; mo: months; pts: patients; NR: not reported; bid: twice daily; d: daily; Obi: Obinutuzumab; C: cycle; D: day; TN: treatment naïve; R/R: relapsed/refractory; m: median.
